# Nanovectorized Microalgal Extracts to Fight *Candida albicans* and *Cutibacterium acnes* Biofilms: Impact of Dual-Species Conditions

**DOI:** 10.3390/antibiotics9060279

**Published:** 2020-05-26

**Authors:** Virginie Lemoine, Clément Bernard, Charlotte Leman-Loubière, Barbara Clément-Larosière, Marion Girardot, Leslie Boudesocque-Delaye, Emilie Munnier, Christine Imbert

**Affiliations:** 1 Laboratoire Ecologie et Biologie des Interactions, Université de Poitiers, UMR CNRS 7267, 86073 Poitiers, France; lemoinev60@gmail.com (V.L.); clement.bernard@univ-poitiers.fr (C.B.); marion.girardot@univ-poitiers.fr (M.G.); 2 Laboratoire SIMBA EA 7502, Faculté de Pharmacie, Université de Tours, 31 avenue Monge, 37200 Tours, France; charlotte.leman-loubiere@clarins.com (C.L.-L.); leslie.boudesocque@univ-tours.fr (L.B.-D.); 3 Dénitral, Groupe COOPERL, 7 rue des Blossières Maroue BP 60328, 22403 Lamballe, France; barbara.clement-larosiere@cooperl.com; 4 Laboratoire Nanomédicaments et Nanosondes EA 6295, Faculté de Pharmacie, Université de Tours, 31 avenue Monge, 37200 Tours, France; emilie.munnier@univ-tours.fr

**Keywords:** antibiofilm, antimicrobial agent, bacteria, fungi, polymicrobial biofilm, microalga, free fatty acids, encapsulation

## Abstract

Biofilm-related infections are a matter of concern especially because of the poor susceptibility of microorganisms to conventional antimicrobial agents. Innovative approaches are needed. The antibiofilm activity of extracts of cyanobacteria *Arthrospira platensis*, rich in free fatty acids, as well as of extract-loaded copper alginate-based nanocarriers, were studied on single- and dual-species biofilms of *Candida albicans* and *Cutibacterium acnes*. Their ability to inhibit the biofilm formation and to eradicate 24 h old biofilms was investigated. Concentrations of each species were evaluated using flow cytometry. Extracts prevented the growth of *C. acnes* single-species biofilms (inhibition > 75% at 0.2 mg/mL) but failed to inhibit preformed biofilms. Nanovectorised extracts reduced the growth of single-species *C. albicans* biofilms (inhibition > 43% at 0.2 mg/mL) while free extracts were weakly or not active. Nanovectorised extracts also inhibited preformed *C. albicans* biofilms by 55% to 77%, whereas the corresponding free extracts were not active. In conclusion, even if the studied nanocarrier systems displayed promising activity, especially against *C. albicans*, their efficacy against dual-species biofilms was limited. This study highlighted that working in such polymicrobial conditions can give a more objective view of the relevance of antibiofilm strategies by taking into account interspecies interactions that can offer additional protection to microbes.

## 1. Introduction

Biofilms are involved in numerous diseases, both superficial and systemic, for instance those affecting the oral cavity, skin or related to an implanted medical device. They can be single species, but most often they are polymicrobial and contain both fungi and bacteria. For example, dermal wounds are colonized by aerobic and anaerobic bacterial and fungal species, most of them belonging to resident microbiota of the surrounding skin, oral cavity and gut, or from the external environment [1]. It has been shown that 60% of chronic wounds exhibit a biofilm which is a major factor in delayed wound healing [2,3,4,5]. Also, it is considered that *Candida* spp. are among the primary causes of delayed healing and infection in both acute and chronic wounds, especially those of a surgical nature [6,7]. Literature data suggest that *Candida* spp. rarely colonize human skin but can cause infection especially in specific conditions such as immune deficiency, diabetes or after antibiotic use [8,9].

Gram-positive bacteria *Cutibacterium acnes* (formerly *Propionibacterium acnes*) [10] are a main colonizer and inhabitant of the skin [11,12] and a good biofilm former, producing both single species and polymicrobial biofilms [13,14,15,16]. Its involvement in chronic skin disease such as acne vulgaris is very well-known and this species is also occasionally involved in non-skin-related infections such as prosthetic joint infections, some of them being related to the formation of a biofilm [17,18]. As for many other microbial species, sessile *C. acnes* cells as well as *Candida albicans* cells have been shown to be more tolerant to conventional antibiotics than their planktonic counterparts [19,20,21].

Our team recently showed that *C. acnes* and *C. albicans* can form dual-species biofilms, with *C. acnes* adhering to both hyphal and yeast forms of *C. albicans* [15]. The presence of metabolically active *C. albicans* cells enhanced the early growth of *C. acnes* under aerobic conditions, while no influence was observed in anaerobic conditions. We also recently demonstrated that the co-presence of these species in biofilms influenced their sensitivity to micafungin, a major conventional antifungal agent. Actually, *C. acnes* was shown to protect *C. albicans* cells from the effect of micafungin in dual-species biofilms [22]. Along the same lines, Montelongo-Jauregui et al. showed that the resistance of *C. albicans* to amphotericin B and caspofungin, as well as the resistance of *Streptococcus gordonii* to clindamycin were increased due to a dual-species biofilm produced by *C. albicans* and with *Streptococcus gordonii*, compared to single-species conditions [23]. Therefore, a double issue should be thus observed—biofilm lifestyle causes itself a decreased susceptibility to antimicrobial agents and the polymicrobial nature of the biofilm can make this lack of susceptibility even worse.

Biofilms are infectious reservoirs and the most effective way to prevent biofilm-related infections requires the eradication of these complex microbial structures, that is their detachment, their disorganization and the killing of all released microbial cells, with these three events needing to be concomitant. Unfortunately, the available antimicrobial conventional molecules fail to reach this challenging goal.

Free fatty acids (FFAs) are physiological antimicrobial agents occurring on skin, exhibiting a wide antimicrobial spectrum (antibacterial, antifungal, antibiofilm…) [24,25]. Microalgae have been well-described as abundant sources of lipids and especially FFAs [26,27]. Those FFAs, especially polyunsaturated (PUFAs), may also represent a potential source of topical drugs against polymicrobial biofilms. Indeed, a previous screening of 29 FFAs based on topical antibacterial activity highlighted that PUFAs were among the most active [25]. *Arthrospira platensis* (formerly *Spirulina platensis*) appeared as a good model among all microalgae as it was the most studied microalgae with a well-known FFA profile.

Due to their lipid nature, FFAs are not able to penetrate the biofilm made of highly hydrophilic exopolysaccharide. Recently, nanosized systems showed their ability to vectorize active molecules in biofilms. Core-shell nanosystems, with a hydrophilic shell and a lipophilic core, seem to be very appropriate vectors for low-polarity active molecules [28,29]. Alginate-based nanocarriers, nanosystems made of a triglyceride core and an alginate gel shell, were shown to be efficient to vectorize FFAs in *C. albicans* biofilm [30]. The reproducibility of the preparation, the stability of the systems and their FDA-approved ingredients constitute key advantages for their use in dermatology. Moreover, they were shown to be stable in dermatological preparations [31].

We previously developed a proof of concept of the potential of *A. platensis* extracts and alginate-based nanocarriers combination as a possible strategy to fight *C. albicans* single-species biofilms. The antibiofilm strategies are all the more innovative and promising in that they are able to act on taxonomically distant and diverse microbial species, because of the polymicrobial nature of most biofilms developing in humans.

Thus, this current study aimed to obtain lipid extracts from Cyanobacteria *A. platensis*, to develop extract-loaded copper alginate-based nanocarriers able to carry a lipid extract and to evaluate the antibiofilm activity of these lipid extracts nanovectorized or free, against both single-species fungal and bacterial biofilms and interkingdom dual-species biofilms.

## 2. Results

### 2.1. A. platensis Extraction

*A. platensis* biomass was extracted using two sustainable solvents—EtOAc and DMC. Resulting extracts were enriched in lipids (Table 1), with a closely related FFA profile. Both extracts contained mainly ω6 PUFA, i.e., linoleic and γ-linolenic acid (more than 60% of total FFAs) (Table 2). Also, lipophilic dyes (chlorophyll and carotenoids) were co-extracted (Table 2), but their content remained low, highlighting again the good selectivity of these solvents towards lipids.

### 2.2. A. platensis Extracts Vectorization

Extract-loaded ANCs were prepared with EtOAc extract and DMC extract. Physicochemical characteristics are shown in Table 3.

Extract-loaded ANCs show similar size and surface potential as empty ANCs with a pure Labrafac^®®^ WL 1349 core. The polydispersity index lower than 0.2 shows a monodispersity of the suspensions, guaranteeing the reproducibility of the dosage. The negative surface charge of the nanocarriers participates to the colloidal stability of the nanocarriers and should not limit their interaction with the biofilms. Indeed, even if biofilms are generally considered negatively charged and could thus bind more easily to cationic nanoparticles [32], several negatively charged systems displayed antibiofilm efficacy [28,33]. The native ANC suspension shows a concentration in *A. platensis* extract of ~1 mg/mL.

### 2.3. Ability of A. platensis Extracts to Prevent Biofilm Formation

**Figure 1:**
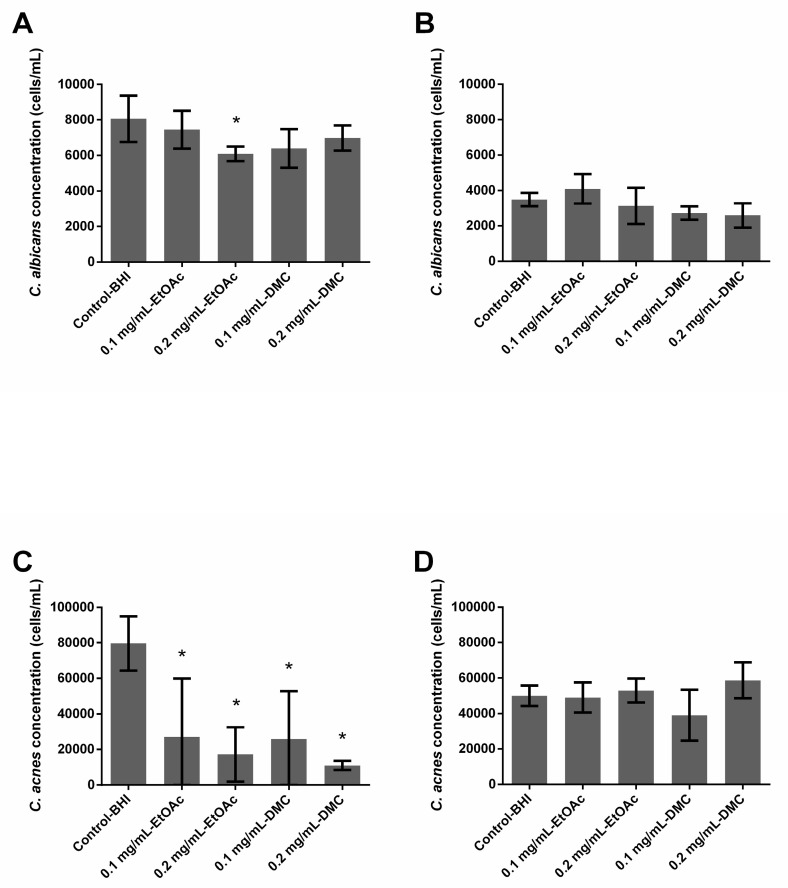
Ability of *Arthrospira fusiformis* extracts to prevent biofilm formation. Single-species biofilms (*C. albicans*) (**A**); *C. albicans* concentration obtained in dual-species biofilms (*C. albicans* + *C. acnes*) (**B**); single-species biofilms (*C. acnes*) (**C**); *C. acnes* concentration obtained in dual-species biofilms (*C. albicans* + *C. acnes*) (**D**). Results are expressed as mean ± SD. * *p* < 0.005: test condition vs. BHI-control (biofilms treated with BHI only).

In single-species conditions, EtOAc extract used at 0.2 mg/mL displayed a significant (*p* = 0.0001) but very limited antibiofilm formation effect against *C. albicans* (24.4% inhibition) (Figure 1A). This extract used at 0.1 mg/mL was not active against *C. albicans*, and DMC extracts (at both 0.1 and 0.2 mg/mL) as well. Both EtOAc and DMC extracts significantly reduced the growth of *C. acnes* biofilms, regardless of the tested concentrations—inhibition ranged between 66.0% and 78.4% (EtOAc extract, *p* ≤ 0.003) and between 67.6% and 86.2% (DMC extract, *p* ≤ 0.0008) (Figure 1C). However, no real conclusion can be made in the case of EtOAc (0.1 and 0.2 mg/mL) and DMC (0.1 mg/mL) as the error bars are very high.

In dual-species conditions, neither EtOAc nor DMC extract solutions were able to reduce the biofilm formation of *C. albicans* and no reduction was observed in the fungal and bacterial populations.

### 2.4. Ability of A. platensis Extracts to Eradicate Preformed Biofilms

**Figure 2:**
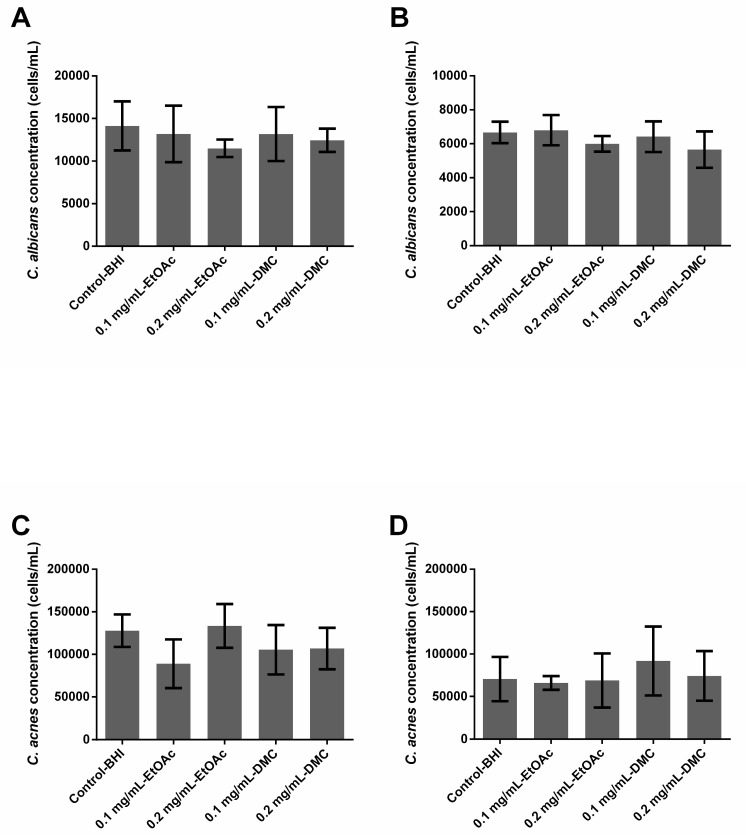
Ability of *Arthrospira fusiformis* extracts to eradicate preformed biofilm. Single-species biofilms (*C. albicans*) (**A**); *C. albicans* concentration obtained in dual-species biofilms (*C. albicans* + *C. acnes*) (**B**); single-species biofilms (*C. acnes*) (**C**); *C. acnes* concentration obtained in dual-species biofilms (*C. albicans* + *C. acnes*) (**D**). Results are expressed as mean ± SD. * *p* < 0.005: test condition vs. BHI-control (biofilms treated with BHI only).

None of the extracts, whatever the tested concentration, had any effect on *C. albicans* or *C. acnes* preformed single-species or dual-species biofilms. No reduction was observed in the fungal and bacterial populations after a 24 h treatment (Figure 2).

### 2.5. Ability of A. platensis Extracts Encapsulated in Alginate-Based Nanocarriers to Prevent Biofilm Formation

**Figure 3:**
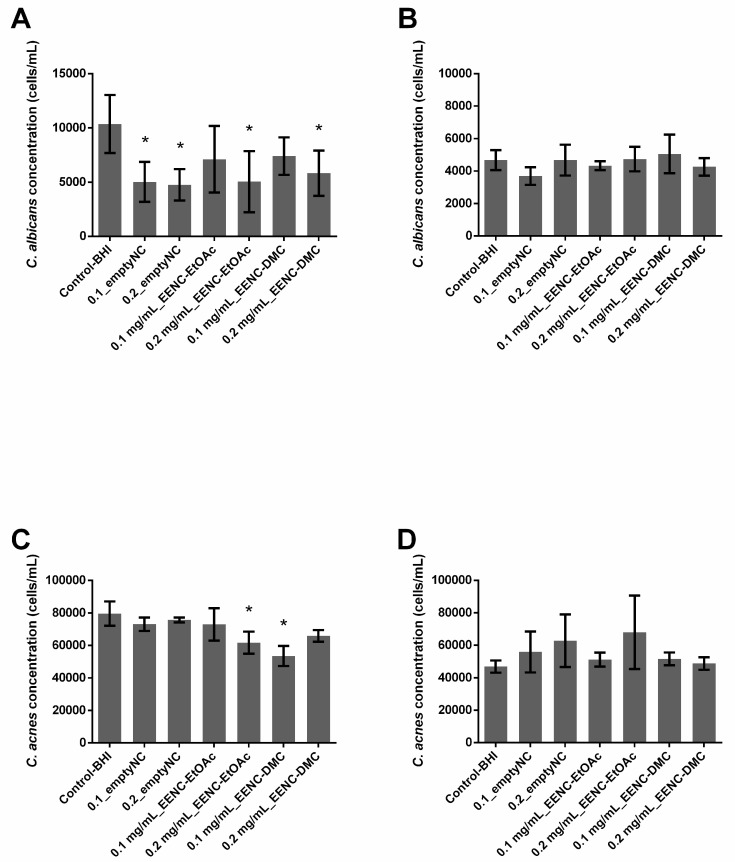
Ability of *Arthrospira fusiformis* extracts encapsulated in alginate nanocarriers to prevent biofilm formation. Single-species biofilms (*C. albicans*) (**A**); *C. albicans* concentration obtained in dual-species biofilms (*C. albicans* + *C. acnes*) (**B**); single-species biofilms (*C. acnes*) (**C**); *C. acnes* concentration obtained in dual species biofilms (*C. albicans* + *C. acnes*) (**D**). Results are expressed as mean ± SD. * *p* < 0.005: test condition vs. BHI-control (biofilms treated with BHI only).

In single-species conditions, empty nanocarriers inhibited the growth of *C. albicans* biofilms by 51.55% (0.1_emptyNC, *p* = 0.001) or 54.14% (0.2_emptyNC, *p* = 0.0002), while they had no effect on *C. acnes* biofilms (Figure 3A,C). Nanocarriers loaded with extract solutions at 0.2 mg/mL (0.2 mg/mL_EENC) inhibited the growth of *C. albicans* biofilms by 51.35% (EtOAc, *p* = 0.0031) or 43.77% (DMC, *p* = 0.0021) while those loaded with extract solutions at 0.1 mg/mL (0.1 mg/mL_EENC) had no significant influence. Regarding the growth of *C. acnes* biofilms, only nanocarriers loaded with EtOAc extract at 0.2 mg/mL and DMC extract at 0.1 mg/mL demonstrated a weak inhibitory activity of 22.48% (*p* = 0.0016) and 32.74% (*p* = 0.0004), respectively (Figure 3C).

In dual-species conditions, nanocarriers loaded with extract solutions did not limit the growth of either *C. albicans* or *C. acnes* in biofilms. Only empty nanocarriers (0.1_emptyNC, *p* = 0.0046) displayed a weak activity but were not significant (*p* > 0.005) against *C. albicans* growth (21.0%) and no reduction was observed on *C. acnes* population (Figure 3B,D).

### 2.6. Ability of A. platensis Extracts Encapsulated in Alginate-Based Nanocarriers to Eradicate Preformed Biofilms

**Figure 4:**
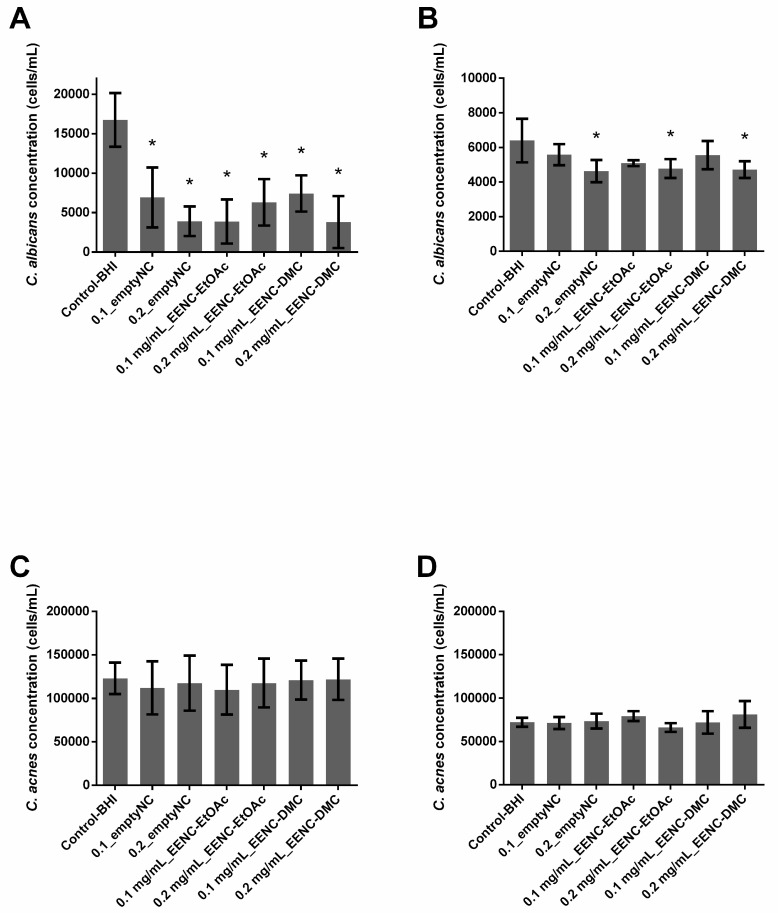
Ability of *Arthrospira fusiformis* extracts encapsulated in alginate nanocarriers to eradicate preformed biofilm. Single-species biofilms (*C. albicans*) (**A**); *C. albicans* concentration obtained in dual-species biofilms (*C. albicans* + *C. acnes*) (**B**); single-species biofilms (*C. acnes*) (**C**); *C. acnes* concentration obtained in dual-species biofilms (*C. albicans* + *C. acnes*) (**D**). Results are expressed as mean ± SD. * *p* < 0.005: test condition vs BHI-control (biofilms treated with BHI only).

In single-species conditions, empty nanocarriers inhibited preformed biofilms of *C. albicans* by 58.7% (0.1_emptyNC, *p* < 0.0001) or 76.69% (0.2_emptyNC, *p* < 0.0001), whereas they had no effect on *C. acnes* biofilms (Figure 4A,B). Whatever the conditions, all nanocarriers loaded with extract solutions inhibited preformed single species *C. albicans* biofilms (*p* < 0.0001) by at least 55%; nanocarriers loaded with EtOAc extracts inhibited biofilms by 76.9% (0.1 mg/mL_EENC-EtOAc) and 62.35% (0.2 mg/mL_EENC-EtOAct) whereas those loaded with DMC extracts induced a 55.69% (0.1 mg/mL_EENC-DMC) and a 77.32% (0.2 mg/mL_EENC-DMC) inhibition. On the contrary, whatever the conditions, both empty and loaded nanocarriers failed to significantly reduce an already formed single-species biofilm of *C. acnes* (*p* > 0.005) (Figure 4C).

In dual-species conditions, empty nanocarriers as well as those loaded with extract solutions induced inhibition always less than 28% of already formed dual-species biofilms, whatever the target population (*C. albicans* or *C. acnes*) (Figure 4B,D). Significant *p*-values demonstrating an inhibition of the *C. albicans* population were only observed in the case of 0.2_emptyNC (inhibition: 27.5%), 0.2 mg/mL_EENC-EtOAc (inhibition: 25.3%) and 0.2 mg/mL_EENC-DMC (inhibition: 26.3%) (Figure 4B).

## 3. Discussion

The results are in accordance with those previously obtained when studying the activity of EtOAc extract at 0.2 mg/mL on *C. albicans* biofilms [30]. The ability of EtOAct extract at 0.2 mg/mL to inhibit *C. albicans* biofilms growth evidenced by the significant decrease in the number of cells forming treated biofilms (FCM approach) (Figure 1A) agrees with previous results showing that this extract was able to reduce the metabolic activity of *C. albicans* forming treated biofilms (XTT method). However, EtOAc extract at 0.1 mg/mL and DMC extract at 0.1 or 0.2 mg/mL did not manage to decrease yeast concentration in biofilms, although they previously showed antimetabolic activity. The XTT method is a classical method used to quantify fungal biofilms [34,35,36]. However, this method does not allow a differentiation between bacterial and fungal populations in dual-species biofilms. That is why the FCM approach used for the current study was recently developed [22,37]. A comparative study previously suggested that results provided by colony-forming unit (CFU) counts, XTT reduction or FCM counts were generally comparable and occasional differences could be explained by the specificity and targets of each method [37]. For example, metabolic activity can be reduced without any change in the cell number explaining some divergence in XTT versus CFU or FCM count results. Slight differences between previous and present results could also be at least partially explained by the fact that two different *A. platensis* biomasses were used in these studies, leading to different compositions of extracts. Growth conditions impact the FFA profile as large amounts of ω6-MUFAs and PUFAs were highlighted here, with decreased rates of saturated FFAs, the latter being known to exhibit higher antifungal activity.

By comparing results obtained from growing biofilms (prophylactic activity) and preformed ones (curative activity), we observed that EtOAc extract at 0.2 mg/mL loses its activity once the biofilm is formed (Figure 1A and Figure 2A). Similarly, although all tested extracts significantly limited the growth of single species *C. acnes* biofilms, they were not active anymore once the biofilm was preformed (Figure 1C and Figure 2C). The extracts, whether free or nanovectorized, were not active against dual-species biofilms, growing or already formed as well (Figure 1C,D and Figure 2B,D). Moreover, since single-species *C. albicans* biofilms were prepared aerobically and those involving *C. acnes* anaerobically, a role of the presence of oxygen could not be excluded to explain the different levels of antibiofilm activity that have been observed. In fact, the mechanism of action of the FFA is not completely elucidated. Some studies suggested that their antimicrobial activity would be partly explained by the formation of PUFA peroxidation products [24], which would be favored in an aerobic environment. These oxidized metabolites would act according to a mechanism different from that of native FFAs [24], explaining the residual activity observed on *C. acnes*. As we could expect, these results suggest that preventing the formation of a biofilm is easier than eradicating this biofilm once it is formed.

Different teams demonstrated that biofilms made of more than one species presented reduced susceptibility to antimicrobial treatment compared to single-species biofilms [38,39]. In addition to studying the activity of the extracts and nanocarriers loaded or not by extracts on single-species biofilms, our work assessed the impact of the dual-species nature of the biofilms. Indeed, our results showed that nanocarriers loaded or otherwise with *A. platensis* EtOAc extracts or loaded or not with *A. platensis* DMC extracts as well significantly reduced both already formed and formation of *C. albicans* single-species biofilms, but displayed no or poor activity against *C. albicans* in dual-species biofilms (Figure 3; Figure 4A,B). These results thus suggest that *C. albicans* growing with *C. acnes* in dual-species biofilms is more difficult to inhibit than in single-species ones, which agrees with previous studies on the efficacy of micafungin against *C. albicans* in these two conditions [22]. More generally, results published in recent years suggest that bacteria and fungi from dual-species biofilms such as *C. albicans–Staphylococcus* spp. or *C. albicans–Streptococcus* spp. often exhibit reduced susceptibilities towards antibiotic or antifungal agents, which is at least partially caused by their synergistic interaction [23,38,40,41,42,43]. This study confirmed the activity of the empty nanocarriers against *C. albicans* biofilms which was already observed by Boutin et al. in 2019 [30], suggesting that copper ions could efficiently reach *C. albicans* cells through this single-species biofilm. Cheong et al., 2020 recently confirmed that copper displayed a high antifungal activity against *C. albicans* [44]. Unfortunately, we observed that empty nanocarriers lose their activity at least partially against *C. albicans* as soon as *C. acnes* is present in biofilms, whatever the age of the studied biofilm. Punniyakotti et al. 2020, recently reported the antibiofilm activity of copper nanoparticles studying *Pseudomonas* and *Staphylococcus* species [45]. They hypothesized that Cu^2+^ ions liberated from the nanoparticles would be engrossed by the bacterial cell surface and cause cell damage, affecting biofilm development. These authors suggested that the surface binding capability of copper ions would play a key role in the biofilm inhibition. Although we can hypothesize a similar mechanism to explain the activity against fungi, there is no clear explanation as to why empty nanocarriers failed to inhibit biofilm in the presence of *C. acnes*. Nanocarriers loaded with *A. platensis* extracts failed to significantly prevent the formation of *C. acnes* biofilms whereas *A. platensis* extracts without nanocarriers did it in the range of 66.0% to 86.2%. As empty nanocarriers display no activity either, we can hypothesize that nanocarrier loading would counteract the action of extracts against these bacteria (Figure 1; Figure 3C). Conversely, the encapsulation of *A. platensis* extracts induced up to 51.35% of inhibition against the formation of *C. albicans* single-species biofilms (Figure 1A and Figure 3A). As the empty nanocarriers inhibited *C. albicans* single-species biofilm formation and eradicated biofilms, the activity cannot be totally attributed to the extracts. Unfortunately, this encapsulation did not allow the growth inhibition of dual-species biofilms, whatever the studied species (Figure 1; Figure 3B,D).

Finally, *A. platensis* extracts alone or encapsulated in nanosystems displayed an absence of activity against *C. acnes* preformed biofilms (Figure 2C and Figure 4C) whereas the encapsulation of *A. platensis* extracts gave a promising activity against *C. albicans* preformed single-species biofilms, inducing inhibition up to 77.32% (Figure 2A and Figure 4A). Whatever the microorganism studied, the encapsulation does not lead to the obtention of an efficient and significant inhibition of preformed dual-species biofilms (Figure 2B,D and Figure 4B,D)

Very few authors compared the effect of nanosystems vectorizing antimicrobial agents on mono- or multispecies biofilms [46,47], and even less on biofilms mixing Gram-positive bacteria and fungi. It is now established that the efficacy of nanosystems on biofilms is linked to their capacity for deeply penetrating the matrix [32]. However, the penetration of nanoparticles into biofilms is highly dependent on the surface characteristics of the nanoparticles [46,48]. Our results suggest that ANCs can diffuse through the extracellular polymeric substance (EPS) of *C. albicans* biofilm, but are not able to diffuse in the EPS of *C. acnes* and in that of polymicrobial biofilm matrix as well. Anjum et al. showed that PLGA nanoparticules loaded with xylitol successfully penetrated into the EPS matrix of single-species biofilms of *S. aureus* or *Pseudomonas aeruginosa*, and also of dual-species biofilms [46]. In the study of Anjum et al., penetration was made easier by adding a ligand onto the nanoparticle surface targeting the biofilm matrix. Tan et al. measured the antibiofilm activity of nanoparticles including enzymes targeting the matrix of biofilms composed of *S. aureus* and *C. albicans* [47]. The particles were able to disrupt in a similar manner single-species or dual-species biofilms, but it was observed that adhesion of bacteria to *Candida* hyphae made their surface less accessible to antimicrobial molecules. This obstacle was already described for free antimicrobial molecules in dual-species biofilms of *S. aureus* and *Fusarium falciforme* [49]. This interaction between the microbial species was also observed for *C. acnes* et *C. albicans* [22] and could participate in the loss of activity of ANCs on *C. albicans* in the dual-species biofilm.

In conclusion, our results highlight the interest of *A. platensis* extracts in preventing the formation of *C. acnes* single-species biofilms. They also suggest that even if the nanocarrier developed by our team offers interesting features, especially in the case of *C. albicans*, its activity against dual-species biofilms is much more limited at the concentrations tested. Even if in vitro models represent simplified models, far from real clinical conditions, developing polymicrobial conditions gives a more realistic representation of clinical biofilms that develop in the human body. This study clearly demonstrated the impact of polymicrobial conditions on the antibiofilm efficacy of nanovectorized antimicrobial systems and highlighted the importance of working in such polymicrobial conditions to have a more objective view of the tested molecules or systems.

## 4. Materials and Methods

### 4.1. Chemicals

Ethyl acetate (EtOAc), methanol (MeOH), toluene, hexane, formic acid, diethylether, glacial acetic acid, petroleum ether, sulfuric acid 96% (H_2_SO_4_) and dimethylsulfoxide (DMSO) were purchased from Carlo Erba (Val de Reuil, France). Dimethyl carbonate (DMC), sodium alginate, (±)-α-tocophérol 96% (vitamin E), oleic acid, linoleic acid, palmitic acid, myristic acid, stearic acid, palmitoleic acid, γ-linolenic acid, 2,3-bis-(2-methoxy-4-nitro-5-sulfophenyl)-2H-tetrazolium-5-carboxanilide (XTT), menadione and glucose were purchased from Sigma Aldrich (Saint-Quentin Fallavier, France). Phosphoric acid 85% was purchased from Merck pro analysis (Darmstad, Germany). Labrafac^®®^ WL 1349 was purchased from Gattefossé (Saint-Priest, France). Montane 80^®®^ and Montanox 80^®®^ were purchased from Seppic (Castres, France). Copper nitrate Cu(NO_3_)_2_ was purchased from Fisher Scientific SAS (Illkirch, France). Vanillin, acetic acid trihydrate 99+% were purchased from Acros Organics (Geel, Belgium). Water was purified using a Milli-Q system (Millipore Corporation, Bedford, MA, USA).

### 4.2. Biomass

*Arthrospira platensis* was cultivated and harvested by DENITRAL SA (Lamballe, France) and kindly provided by Dr Barbara Clément-Larosière.

### 4.3. Extraction Protocol and Extracts Analyses

A total of 1 g of biomass was extracted with DMC or EtOAc according to the protocol described by Boutin et al. [30]. The calibration curve was built up using castor oil and results were expressed as mg of equivalent of castor oil in the extract.

Pigments and total lipid rates were obtained using protocol described in Boutin et al. (2019) [30]. FFA profiles were obtained using the LC-ESI-MS protocol adapted from Samburova et al. (2013) [50]. Briefly, LC-ESI-MS analyses were performed on an Acquity H-Class with an SQD detector (Waters, Saint Quentin en Yvelines, France). The system was fitted with a BEH C18 (50 × 2.1 mm; 1.7 µm particle size). The column oven was set at 40 °C. Mobile phases were A Water 0.1% NH_3_ aq; B acetonitrile 0.1% NH_3_ aq. Flow rate was 0.25 mL/min and the gradient was set as follows—initial solvent B content was 10%, raised to 40% in 2 min, 90% in 23 min and 100% in 1 min and maintained for 9 min. ESI in negative mode was performed with cone voltage set at 50 V and capillary voltage at 2.8 kV.

### 4.4. Alginate-Based Nanocarriers Preparation and Characterization

Alginate-based nanocarriers (ANCs) were prepared using ultrasound oil-in-water emulsification followed by surface gelation with cupric ions inspired by Nguyen et al. [31] and adapted by Boutin et al. [30]. Briefly, an *A. platensis* lipid extract solution in Labrafac ^®^ WL 1349 (6 mg/mL) was emulsified with a sodium alginate solution in presence of nonionic surfactant, using an ultrasonic probe (Vibra-cell ultrasonic processor, Sonics, Newtown, CT, USA, 20 kHz). The resulting nanoemulsion was mixed under ultrasounds stirring with a solution of copper ions, which complex alginates to form an insoluble copper-alginate gel at the surface of the nanodroplets.

The hydrodynamic diameter and polydispersity index (PdI) of the ANC aqueous suspensions were measured using a dynamic light scattering (DLS) instrument (NanoZS, Malvern Panalytical, Malvern, UK). Each sample was diluted 1:50 in ultrapure water before measurements. Zeta potential was determined on the same sample with the same instrument. Measurements were made in triplicate at 25 °C.

### 4.5. Bacterial and Fungal Organisms

*C. albicans* ATCC^®^ 28367™ and *C. acnes* ATCC^®^ 6919 were used for this study.

Yeasts were cultured on Sabouraud Glucose with Chloramphenicol agar plates aerobically at 37 °C whereas *C. acnes* was cultured on Brain Heart Infusion (BHI) agar plates supplemented with 10% of defibrinated horse blood anaerobically at 37 °C. Before biofilm experiments, *C. albicans* and *C. acnes* were cultured overnight in BHI at 37 °C in aerobic and anaerobic conditions, respectively. Following incubation, cultures were washed with PBS (centrifugation at 2000× *g*, 10 min) and adjusted to 2 × 10^7^ cells/mL and 2 × 10^8^ cells/mL in fresh BHI for *C. albicans* and *C. acnes* respectively.

### 4.6. Antibiofim Formation Assay

Single-species *C. albicans*, single-species *C. acnes* and polymicrobial *C. albicans*-*C. acnes* biofilms were formed in 96-wells flat bottom nontreated polystyrene microplates. In the single-species condition, wells received 100 µL of microbial suspensions. In polymicrobial condition, wells received 50 µL of both microbial suspensions.

Antibiofilm formation activities of lipid extracts previously dissolved in DMSO were tested at two concentrations—0.1 and 0.2 mg/mL. Final DMSO concentrations did not exceed 2% of the overall volume in wells. For extracts included in nanocarriers (NCs), nanosystem tested concentrations were chosen to display extracts at 0.1 and 0.2 mg/mL in ultrapure water—“extract equivalent in nanocarrier” (mg/mL_EENC). Finally, empty nanocarriers were tested as controls and the studied concentrations corresponded to those present in nanocarriers loaded with extracts at 0.1 and 0.2 mg/mL (0.1_emptyNC and 0.2_emptyNC). A total of 100 µL of extract or nanosystem solutions diluted in BHI were then added to the wells. Some wells without extract or nanosystem solution were reserved as a control and received 100 µl of fresh BHI (BHI control). Microplates containing only *C. albicans* were incubated 24 h at 37 °C in aerobic conditions while microplates containing *C. acnes* or both microorganisms were incubated in anaerobic conditions.

After incubation, cell concentrations were determined using a protocol adapted from the work of Kerstens et al., 2015 [51]. Planktonic cells were eliminated (2 rounds of washing with 200 µL of PBS) and sessile cells were scraped off from the microplate bottom using sterile tips. An extensive rinsing of the microplate bottom was performed to detach remaining microorganisms. The obtained suspensions were sonicated for 10 min to break down aggregates (Elmasonic S 30, Elma Electronic, Wetzikon, Switzerland, 37 Hz). This procedure has no effect on both *C. albicans* and *C. acnes* viability according to literature data [18,51].

In these microbial suspensions, cells concentrations were determined using flow cytometry (FCM). For dual-species conditions, FCM allowed us to distinguish the yeast population from that of bacteria according to their respective sizes and morphologies [22]. Measurements were performed on a CytoFLEX (Beckman Coulter, Indianapolis, IN USA) managed by CytExpert 2.0.0.153 software (Beckman Coulter, Indianapolis, IN, USA) and equipped with a blue laser (λ_ex_ = 488 nm) and a 488/8 bandpass filter. The flow rate used was 30 µL·min^−1^.

### 4.7. Anti-Preformed Biofilm Assay

Single-species *C. albicans*, single-species *C. acnes* and polymicrobial *C. albicans*-*C. acnes* biofilms were formed in 96-well flat-bottom nontreated polystyrene microplates. In single-species condition, wells received 100 µL of microbial suspensions. In polymicrobial condition, wells received 50 µL of both microbial suspensions. Final volume was adjusted to 200 µL using fresh BHI in all conditions. Microplates containing only *C. albicans* were incubated 24 h at 37 °C in aerobic conditions whereas microplates containing *C. acnes* or both microorganisms were incubated in anaerobic conditions.

After incubation, supernatants were removed, and biofilms were carefully rinsed twice with 200 µL of PBS. A total of 100 µL of fresh BHI was added to all wells. Then, wells received 100 µL of extract or nanosystem solutions diluted in BHI. Tested conditions were similar to those presented for antibiofilm formation assays. Some wells without extract or nanosystem solution were reserved as a control. A total of 100 µL of fresh BHI was also used in control wells. Microplates containing only *C. albicans* were incubated 24 h at 37 °C in aerobic conditions whereas microplates containing *C. acnes* or both microorganisms were incubated in anaerobic conditions.

After incubation, cell concentrations were determined as described previously for the antibiofilm formation assay.

### 4.8. Statistical Analysis

Experiments were performed at least in duplicate with four replicates for each condition. Mann–Whitney *U* test was applied to determine statistical significance of the differences between the groups using GraphPad Prism^®^ version 6.01 (GraphPad Software Inc, San Diego, CA USA). Differences were considered significant if *p* < 0.005.

## Figures and Tables

**Table 1:** *A. platensis* extracts composition (total lipids, chlorophylls, carotenoids).

Extract	EtOAc	DMC
**Total lipids (mg of equiv. castor oil/g of extract)**	1115.1 ± 87.2	980.3 ± 67.9
**Chlorophylls (mg/g of extract)**	82.0 ± 8.1	52.6 ± 2.5
**Carotenoids (mg/g of extracts)**	53.5 ± 8.1	61.0 ± 2.6

Data are shown as mean ± SD; *n* = 3.

**Table 2:** Free fatty acid (FFA) ratios in *A. platensis* extracts, in relative percentage of total FFA.

	EtOAc	DMC
**Saturated**		
Myristic acid	nd	nd
Palmitic acid	27.0%	21.9%
Stearic acid	5.1%	5.7%
**MUFA**		
Myristoleic acid	nd	nd
Palmitoleic acid	nd	nd
Oleic acid	4.8%	4.8%
**PUFA**		
Linoleic acid	41.1%	42.3%
γ-Linolenic acid	22.0%	25.2%

nd = nondetected; *n* = 1.

**Table 3:** Physicochemical characteristics of extract-loaded alginate-based nanocarriers.

	Hydrodynamic Diameter (nm) (Mean ± SD, *n* = 3)	Polydispersity Index (Mean ± SD, *n* = 3)	Zeta Potential (mV) (Mean ± SD, *n* = 3)
Empty ANC	249 ± 9	0.129 ± 0.044	−24.5 ± 0.7
EtOAc extract-loaded ANC	236 ± 2	0.147 ± 0.018	−24.2 ± 0.2
DMC-extract loaded ANC	250 ± 1	0.139 ± 0.015	−24.1 ± 0.9
